# *Chiro*surveillance: The use of native bats to detect invasive agricultural pests

**DOI:** 10.1371/journal.pone.0173321

**Published:** 2017-03-29

**Authors:** Brooke Maslo, Rafael Valentin, Karen Leu, Kathleen Kerwin, George C. Hamilton, Amanda Bevan, Nina H. Fefferman, Dina M. Fonseca

**Affiliations:** 1 Department of Ecology, Evolution and Natural Resources, Rutgers, The State University of New Jersey, New Brunswick, NJ, United States of America; 2 Rutgers Cooperative Extension, New Jersey Agricultural Experiment Station, Rutgers, The State University of New Jersey, New Brunswick, NJ, United States of America; 3 Department of Entomology, Rutgers, The State University of New Jersey, New Brunswick, NJ, United States of America; 4 Ecology and Evolutionary Biology, The University of Tennessee, Knoxville, TN, United States of America; University of Crete, GREECE

## Abstract

Invasive insect pests cost the agricultural industry billions of dollars annually in crop losses. Timely detection of pests is critical for management efficiency. Innovative pest detection strategies, such as environmental DNA (eDNA) techniques, combined with efficient predators, maximize sampling resolution across space and time and may improve surveillance. We tested the hypothesis that temperate insectivorous bats can be important sentinels of agricultural insect pest surveillance. Specifically, we used a new high-sensitivity molecular assay for invasive brown marmorated stink bugs (*Halyomorpha halys*) to examine the extent to which big brown bats (*Eptesicus fuscus*) detect agricultural pests in the landscape. We documented consistent seasonal predation of stink bugs by big brown bats. Importantly, bats detected brown marmorated stink bugs 3–4 weeks earlier than the current standard monitoring tool, blacklight traps, across all sites. We highlight here the previously unrecognized potential ecosystem service of bats as agents of pest surveillance (or *chiro*surveillance). Additional studies examining interactions between other bat and insect pest species, coupled with comparisons of detectability among various conventional monitoring methods, are needed to verify the patterns extracted from this study. Ultimately, robust economic analyses will be needed to assess the cost-effectiveness of *chiro*surveillance as a standard strategy for integrated pest management.

## Introduction

Agricultural insect pests cost the U.S. agricultural industry $33 billion per year in crop losses [1]. Timely detection of insect pest irruptions is critical for effective integrated pest management (IPM) because management efficiency decreases exponentially with time since detection [2]. In the case of emerging invasive insect pests, prompt detection prior to establishment is critical, but the sampling effort required may be logistically or economically infeasible given the rarity of the target pest in the landscape. Developing innovative cost-effective strategies that maximize sampling resolution across space and time may improve detectability, and ultimately control of these pests.

Modern molecular methods have now made it possible to detect highly degraded DNA of invasive species from environmental samples, termed environmental DNA (eDNA; e.g., [3]). The primary advantages of eDNA techniques are that they can detect the presence of a target species, even when it is rare (i.e. early in the invasion or infestation), better than conventional survey methods [4, 5]. To date, eDNA has been used primarily in aquatic ecosystems to detect both invasive and rare species by collecting water samples from suspect water bodies, concentrating all the eDNA present into a secondary medium, then extracting and testing the medium for the presence of target species’ eDNA with specific assays (e.g., invasive asian carp in the Great Lakes, USA [4, 6]). In addition, eDNA techniques have also been used to identify pathogens in species of conservation concern [7], and in gut content and fecal analysis. Gut content and fecal analysis of terrestrial fauna has focused primarily on dietary studies (e.g., [8, 9, 10]) or been used to identify imperiled species and monitor their population trends (e.g., [11, 12]). Here we propose a new application of eDNA analytical techniques and lay the groundwork for the innovative use of highly mobile generalist vertebrate predators for effective invasive species surveillance in terrestrial systems.

Due to their high foraging efficiency, range and diverse diet, temperate insectivorous bats consume many different species of invertebrates [13, 14]. The foraging range of bats is expansive [15], but they return to the same roost site nightly, making routine sampling feasible. Therefore, bats may be important sentinels of insect pest outbreaks if: 1) they consume insect pests while at low densities, and 2) we can confirm reliably insect species in bat guano earlier than in standard monitoring traps.

We tested this concept by examining the extent to which big brown bats (*Eptesicus fuscus*) detect brown marmorated stink bugs (BMSB; *Halyomorpha halys*; Hemiptera: Pentatomidae) in agricultural landscapes. BMSB is a recently introduced, severe agricultural pest that is active in the northeastern US from May through September [16] and survives harsh winters by exploiting human-made structures (e.g., sheds, barns, homes) [17]. Following their introduction in Pennsylvania from Asia in the mid to late 1990’s, they have been recorded to feed upon a wide variety of fruits, vegetables, field crops and ornamental plants [17] and can cause significant economic damage [18]. In its core invasive range in the U.S., BMSB typically have 1–2 generations per year, with non-reproductive BMSB adults overwintering in both natural and man-made sites [17]. During the reproductive season, BMSB are active both day and night and appear capable of traveling ~2km within a 24-hr period [17].

Current BMSB monitoring is primarily conducted through blacklight traps placed within agricultural fields [19], where BMSB aggregate to feed during spring and summer. Blacklight traps capitalize on the affinity of night-flying insects for light sources; however, BMSB response to light stimuli varies across the growing season [17]. Particularly in late summer, BMSB respond more aggressively to aggregation pheromones emitted by other individuals at overwintering sites than to light. In addition, the cone of attraction of blacklight traps is small (2-100m) attracting only individuals in close proximity of the traps [20]. In early spring, BMSB adults emerging from hibernation typically stay in hardwood trees and forests, entering the crop fields only when they are ready to lay eggs [21]. Therefore, blacklight trap catches may significantly miss or under-represent BMSB occurrence in the early and late growing season.

A sampling strategy that eliminates the need for visual identification of target species and lowers the detection threshold for target species can significantly reduce sampling effort and increase sampling precision. Big brown bats develop summer colonies in barns and other manmade structures often used as overwintering sites by BMSB [17], and they forage in a variety of open and forested habitat types. In particular, female big brown bats exhibit site fidelity to foraging areas, consistently remaining within a ~5-km radius of their roost site where they raise their pups [22, 23]. Using a newly developed high-sensitivity molecular assay to identify BMSB DNA remnants, we document consistent seasonal predation on this species by big brown bats. We compare the threshold of detectability of BMSB between bat guano samples and standard monitoring practices (black light traps) and compare the potential effectiveness of these two monitoring strategies.

## Materials and methods

**Fig 1:**
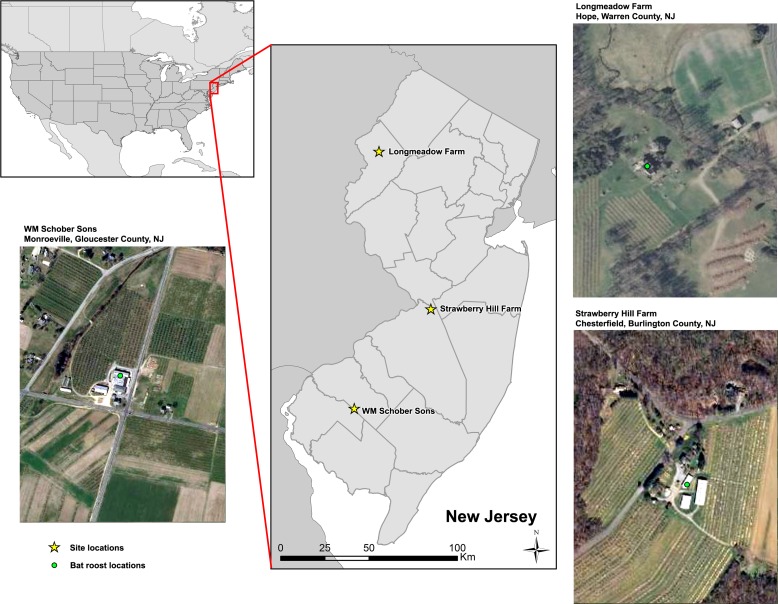
**Location of (A) Longmeadow Farm, (B) Strawberry Hill Farm, and (C) WM Schober Sons farm within New Jersey, USA.** Roosts were located in or immediately adjacent to fruit tree orchards.

We conducted our study in three tree fruit orchards in New Jersey, USA, each with a maternity colony of big brown bats present during the spring and summer of 2013. Longmeadow Farms, located in Hope Township, Warren County, NJ had ~30 bats roosting in the attic of the farmhouse approximately 25 m from the orchard. Strawberry Hill Farm, located in Chesterfield Township, Burlington County, NJ had ~100 bats occupying four roost boxes within 45 m of the orchard, and WM Schober Sons Orchard, in Monroeville Township, Gloucester County, NJ had ~100 bats residing in the loft of the farm’s packing house, approximately 50 m from the orchard (Fig 1). All study sites were privately owned agricultural lands, and we obtained approval from landowners to conduct our field investigation. Colony sizes at each site were estimated by 1–2 observers standing ~30m from each access point of the roost and counting individuals as they exited. We performed the census twice during the season (late May and late July). Counts occurred in dry weather conditions with little to no wind and lasted from 30 minutes prior to sunset until 10 minutes after the last bat exited (e.g., [24, 25, 26]).

On a weekly basis between April 2 –September 18, we collected 100 individual guano pellets (each ~7 mm in length) from beneath bat roosts at each orchard ~1 hr before sunset (total of 60 weekly samples across all sites). To increase the likelihood that our sample represented the digestive output of multiple bats, we selected pellets from all guano piles present at the time. During guano collection we did not handle bats, nor did we initiate or observe any evidence of disturbance of the bat colonies. We placed all the pellets collected from a roost into carefully labeled sterile 50 mL plastic specimen jars (Carolina Biological Supply Company, Burlington, NC, USA) taking strict measures to minimize the loss of integrity of guano pellets by keeping them loosely packed and dry. After guano collection, we swept the remaining guano away to ensure that guano pellets collected the following week were representative of the new week. We transported samples to a laboratory and placed them at -20°C while they awaited analysis.

From each weekly sample, we randomly subsampled and pooled approximately 16–20 pellets for analysis, based upon species accumulation-sampling intensity relationships generated in previous analyses (e.g., [27]). This sampling intensity and nested strategy was chosen to increase the probability that we would detect BMSB if they were consumed, while also decreasing the probability that we would oversample from an individual bat’s single feeding bout. Because big brown bats typically complete two feeding bouts per night, producing ~14 guano pellets per bout [28], we made the very conservative assumption that all pellets generated from an individual bat’s feeding bout might include BMSB DNA if a single individual of that species was consumed.

We pulverized each group of pellets using a TissueLyser (Retsch MM301, Qiagen Sciences, Germantown, MD, USA) fitted with metal beads to generate a homogenous sample representative of the gut components of multiple bats. Once homogenized, we used a Corning single use v-scoop spatula (Sigma-Aldrich Corp., St. Louis, MO) to sample four scoops (approximately 38±1 mg) into a 2-ml eppendorf tube for extraction. We extracted whole genomic DNA from the four scoop samples with a QiaAmp fast DNA stool mini kit (Qiagen Sciences, Germantown, MD, USA), re-suspended the DNA with 200μl of Qiagen buffer ATE, then measured the amount of detectable DNA with a QuBit dsDNA HS assay kit (Life Technologies, Eugene OR, USA). To avoid contaminating guano samples with BMSB DNA in the lab, we adhered to general eDNA precautions [29] for extraction of DNA by using a dedicated DNA extraction laboratory away from the main lab, with water extractions as negative extraction controls.

To detect traces of BMSB DNA among the DNA extracted from the guano we employed a high-sensitivity real-time PCR assay specific to BMSB [30]. Briefly, we performed real-time PCR reactions in replicates of two using HPCL purified primers for BMSB (BMITS1F – 5'-CGAGGCCGCCGATGA-3'; BMITS1R - 5'-CCCACGAGCCGAGTGATC-3'), which were designed in conjunction with a species specific fluorescent probe (BMITS1TM– 5'-CAGGCAATGAAGCACA-3') targeting a conserved region of the rDNA internal transcribed spacer 1 (ITS1). We created 6 base-10 serial dilutions of a BMSB DNA extraction ranging from 1 ng/ml to 10 fg/ml, which were used as real-time PCR standards and positive controls, as well as for DNA sensitivity tests for the assay. Full validation details of the BMSB assay are described in Valentin *et al*. [30]. We ran reactions at an initial denaturing step of 96°C for 10 minutes, followed by 45 cycles of denaturing at 96°C for 15 seconds and annealing and extension at 60°C for 1 minute. All reactions were run on an Applied Biosystems 7500 Real-Time PCR System (Applied Biosystems, Life Technologies, Carlsbad, CA). Standard curves with slopes within 10% efficiency (i.e. between -3.58 and -3.10) were considered acceptable, while analyses falling outside those bounds were rerun for consistency.

We assessed the average BMSB per hectare at each farm site across the 7 nights prior to each guano sample collection using a network of 110-V blacklight traps maintained by the Rutgers Cooperative Extension (RCE) Vegetable Integrated Pest Management Program (IPM). A total of 60 traps were deployed from May 1 –October 1 on agricultural lands across NJ, spaced approximately 8–20 km apart. Trained IPM scouts checked the traps daily, identified specimens to genus or species (depending on the quality of the specimen), and recorded the total number of individuals of each species present. We mapped trap locations with ArcMap 10.2 (ESRI, Redlands, CA). We used an inverse distance weighted (IDW) algorithm to interpolate the average density of BMSB (individuals/ha) at each farm, based upon the BMSB catch data from each of the nearest 12 blacklight traps.

We compared the dates of detection of BMSB between bat guano and blacklight traps. We also used binary logistic regression to investigate the relationship between BMSB detections in guano and site, insect density, and week of the growing season.

## Results

**Fig 2:**
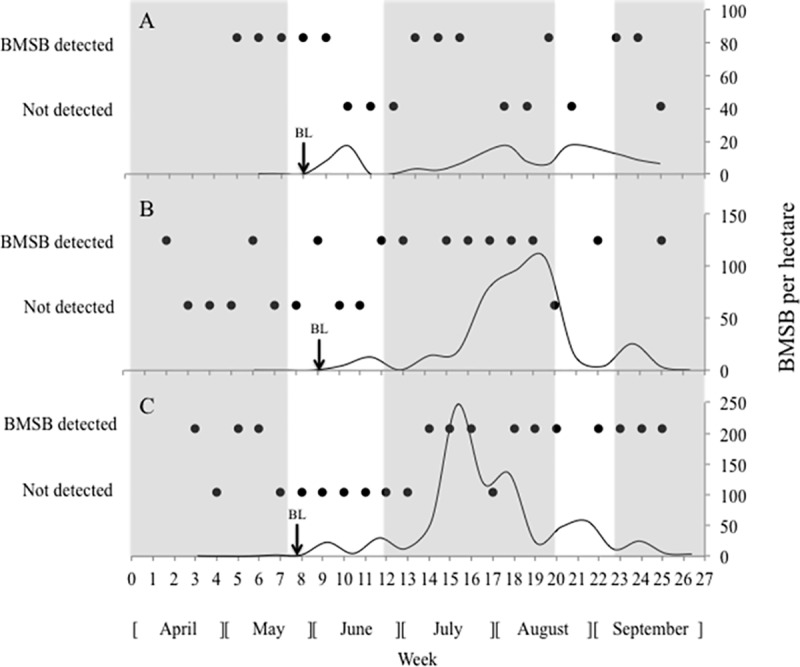
**Weekly big brown bat (*Eptesicus fuscus*) guano samples from (A) Longmeadow Farm, (B) Strawberry Hill Farm, and (C) WM Schober Sons testing positive and negative for brown marmorated stink bug (*Halyomorpha halys*; BMSB). The** secondary axis depicts seasonal BMSB density at each site. Shaded areas indicate BMSB spring emergence from hibernation (Weeks 4–8), peak summer adult activity (Weeks 12–20), and fall aggregation at hibernation sites (Weeks 23–27). Arrows indicate weeks of first BMSB detection in blacklight traps.

The density of BMSB based on blacklight trap monitoring ranged from 0–247 individuals per hectare and remained generally low (<50 individuals per hectare) throughout the early and late season. In the two southern sites, BMSB density significantly increased between mid-June and mid-August, when it reached >100 individuals per hectare (Fig 2). BMSB was first detected in black light traps at WM Schober Sons the week of May 16–22, and at Longmeadow and Strawberry Hill Farms the week of May 24–30.

The detectable DNA from total genomic DNA extracts varied widely from 0.1 ng/μl to 10 ng/μl measured, as expected from samples with DNA in various degrees of degradation. Nonetheless, 60% of the pooled guano sample extractions (36/60) tested positive for BMSB DNA (Fig 2; S1 Table). Of note, both extraction negative controls and qPCR experimental negative controls consistently tested negative for BMSB, indicating we were able to avoid contamination. In contrast to the information from the blacklight traps that showed BMSB occurring for the first time much later in the season, BMSB was first detected in bat guano from Longmeadow, Strawberry Hill, and WM Schober Sons Farms the weeks of April 26 –May 2, April 3–9, and April 10–16, respectively. Positive detections in pooled samples occurred throughout the season and were not correlated with site (p = 0.93), BMSB density estimated from the blacklight traps (p = 0.20) or week of the season (p = 0.23).

## Discussion

Our results demonstrate that big brown bats recognize BMSB, a recent NJ arrival but already an important agricultural pest, as a prey item that can be considered a common dietary component across the season. Two factors potentially forged the pathway for big brown bats to adopt this invasive species as prey. The robust skulls and powerful jaws of big brown bats make them well-adapted for consuming hard-bodied insects, particularly coleopterans and hemipterans (Agosta 2002), and they regularly consume native stink bugs [31, 32].

Critically, big brown bats detected BMSB three to four weeks earlier than blacklight traps across all sites. Although we cannot make comparisons prior to deployment of traps (May 1), it is important to point out that at two sites BMSB was first detected in bat guano by the 2^nd^ week of April. The early detection of BMSB in bat guano highlights the weaknesses in conventional monitoring methods. Currently four types of sampling procedures are available to sample BMSB populations: Blacklight traps, pheromone traps, timed visual counts, and sweep/beat sampling [16, 17]. Blacklight traps represent fixed survey points with relatively low ranges of detection, so they can only attract individuals in the immediate surroundings (the orchards, in this case). Pheromone traps, like blacklight traps, also have a relatively low range of detection and only detect individuals in the proximate area. Time visual counts are used to sample a variety of insect pests in tree fruit and can be used to monitor BMSB. However, the accuracy of this method is dependent on the time of day, the capability of the observer, and the time of the season in which the counts are made. Sweep net and beat sampling methodologies have limitations because their implementation results in the dislodgement of harvestable fruit. In contrast, bats have an extensive sampling radius (5km [33]) and can forage across multiple habitats and potentially across multiple small farms. Early detection of BMSB may be of critical importance to the peach industry because BMSB adults enter peach orchards at or just after bloom to feed on the developing fruit, creating the majority of the economic damage [34]. They also stay in the orchard season long feeding, developing and laying eggs. Detections occuring during the bloom period may provide justification for targeted pesticide application immediately after petal fall to minimize damage. Of note, while *chiro*surveillance, due to the large foraging radius of big brown bats, may not indicate BMSB on a particular farm. However, any positive detection should signal to the farmer the need for careful farm-wide surveillance with visual counts or pheromone traps or in the near future, or the use of enhanced BMSB specific eDNA approaches examining local soil or water samples (Valentin *et al*., unpublished data).

Our work highlights the unrecognized and underexploited potential role of bats as agents of insect pest surveillance, which may have important implications for sustainable agriculture in a global context. Big brown bats are widely distributed across North and Central America, as well as northwestern South America and parts of the Caribbean [35]. Both their affinity for roosting in manmade structures (buildings, bat boxes), as well as their wide foraging range, makes them ideal sentinels for insect pest outbreaks in agricultural settings. Although we focused on a single bat species for this research, it is important to note that global diversity of insectivorous bats is vast [36], markedly increasing the potential applicability of *chiro*surveillance as a useful pest management strategy.

The identification of a new ecosystem service provided by bats increases justification for their conservation at a time when multiple significant threats exist. Across North America, many bat populations have declined precipitously from the fungal disease, white-nose syndrome [37–39]. Other species, particularly migratory bats, are highly vulnerable to mortality associated with wind turbines [40–42]. In addition, persecution of bats (i.e. culling, roost destruction) as a management strategy against zoonotic diseases also threatens bats globally [43–45]. As a pest detection method, *chiro*surveillance offers incentives for stewardship of bat colonies in agricultural landscapes, both through the provisioning of artificial roost sites as well as maintaining non-crop bat habitat (i.e. woodlots, wetlands) as an integral component of the foraging landscape mosaic.

Although using bats for insect pest detection appears promising, additional work examining the effectiveness and feasibility of *chiro*surveillance is needed before application at large scales. Insectivorous bats are generalists, and prey selection is poorly understood. The insect community within the foraging radius of a bat roost changes based upon both spatial (i.e. landscape matrix) and temporal factors (i.e. time of year). How bats’ foraging preferences respond to changes in the available insect community may influence the detection rate of target insect pests. In addition, the breadth of insect pests that can be detected by *chiro*surveillance is not known. We examined the capability of a single bat species to detect a specific insect pest. Studies of predation of multiple insect pests by additional bat species, coupled with comparisons of detectability among the many conventional insect pest monitoring methods (e.g., pheromones, synthetic attractants, crop scouts), are needed to verify the patterns extracted from this study.

In addition, big brown bats are an ideal species from which to collect guano. They are commonly found in manmade structures, making guano highly visible and easy to collect. Guano in artificial roosts (i.e. barns, attics) also is often protected from poor weather conditions, extending the shelf life of the DNA to be extracted. Other human-tolerant cavity-dwelling bat species’ roosts may also serve as consistent sampling sources, whereas roosts of tree-dwelling species may be less reliable because guano may be inconspicuous or inaccessible, and may degrade quickly due to ambient conditions.

Given the expansive foraging radius of big brown bats, it is important to note that BMSB, and thus other potential targets surveyed via *chiro*surveillance, may have been preyed upon from beyond the boundaries of the orchards where the guano was collected, resulting in site specific false positives. These results, when taken alone, may incur added cost to farmers that choose to take action based on these detections, resulting in management action (e.g. spraying) being implemented unnecessarily. However, *chiro*surveillance can be highly informative as part of a nested eDNA approach for making early decisions regarding where to focus surveillance and management actions on a kilometer-wide scale, as these area-wide results are more expansive than current monitoring techniques (e.g. blacklight traps, pheromone traps, etc.). From these data, more fine-scale eDNA surveillance techniques can be implemented on specific farms (Valentin *et al*., unpublished data).

Finally, robust economic analyses are needed to determine the cost-effectiveness of *chiro*surveillance as a standard monitoring tool. Molecular assays can provide superior detection rates for target species relative to standard monitoring protocols [46, 47], but the expense of eDNA techniques may reduce their utility in practice [48]. Detailed investigations of the man-hours and expenses required under both conventional and eDNA strategies can provide useful information for determining the most practical circumstances in which to use them. The preliminary work described here lays an important foundation for understanding the full potential of *chiro*surveillance as an insect monitoring tool and its adoption into standard agricultural pest and invasive species management.

## Supporting information

S1 Table Real-time PCR results for pooled guano samples, and corresponding weekly *Halyomorpha halys* densities, collected in fruit tree orchards in New Jersey, USA, 2013. (DOC)
