# Supplementary material for: Chirosurveillance: The use of native bats to detect invasive agricultural pests
Source: PLoS One. 2017 Mar 29;12(3):e0173321. doi: 10.1371/journal.pone.0173321 (PMC5371280; doi:10.1371/journal.pone.0173321)
Supplement: S1 Table — (DOC) [file pone.0173321.s001.doc]

SUPPLEMENTAL INFORMATION

Table S1. Real-time PCR results for pooled guano samples, and corresponding weekly *Halyomorpha halys* densities, collected in fruit tree orchards in New Jersey, USA, 2013.

|  |  | Pooled Sample | | |  |
| --- | --- | --- | --- | --- | --- |
| Sample | Sample date | | +/- | C_t_ value | *H. halys* density per hectare* |
|  |  | | | | |
| 1 | 5/2 | | + | 28.1 |  |
| 2 | 5/9 | | + | 27.1 | 0.0 |
| 3 | 5/16 | | + | 28.0 | 0.0 |
| 4 | 5/24 | | + | 27.2 | 0.1 |
| 5 | 5/30 | | + | 32.3 | 8.0 |
| 6 | 6/6 | |  |  | 17.0 |
| 7 | 6/13 | |  |  | 0.0 |
| 8 | 6/20 | |  |  | 0.2 |
| 9 | 6/27 | | + | 29.2 | 3.2 |
| 10 | 7/3 | | + | 27.1 | 2.3 |
| 11 | 7/11 | | + | 30.1 | 6.3 |
| 12 | 7/25 | | + |  | 17.5 |
| 13 | 8/1 | |  |  | 7.8 |
| 14 | 8/8 | | + | 32.1 | 6.2 |
| 15 | 8/15 | |  |  | 17.8 |
| 16 | 8/29 | | + | 36.4 | 12.5 |
| 17 | 9/5 | | + | 31.6 | 8.6 |
| 18 | 9/12 | |  |  | 6.4 |
|  |  | | | | |
| 19 | 4/9 | | + | 33.6 |  |
| 20 | 4/16 | |  |  |  |
| 21 | 4/25 | |  |  |  |
| 22 | 5/1 | |  |  |  |
| 23 | 5/8 | | + | 28.0 | 0.0 |
| 24 | 5/15 | |  |  | 0.0 |
| 25 | 5/23 | |  |  | 0.0 |
| 26 | 5/29 | | + | 33.6 | 4.4 |
| 27 | 6/5 | |  |  | 12.8 |
| 28 | 6/12 | |  |  | 0.4 |
| 29 | 6/19 | | + | 27.1 | 14.0 |
| 30 | 6/25 | | + | 23.7 | 18.1 |
| 31 | 7/10 | | + | 30.5 | 76.8 |
| 32 | 7/17 | | + | 26.2 | 96.3 |
| 33 | 7/24 | | + | 31.2 | 108.2 |
| 34 | 7/31 | | + | 28.3 | 15.3 |
| 35 | 8/7 | | + | 28.4 | 4.0 |
| 36 | 8/14 | |  |  | 25.6 |
| 37 | 8/28 | | + | 29.8 | 3.6 |
| 38 | 9/18 | | + | 33.4 | 0.2 |
|  |  | | | | |
| 39 | 4/16 | | + | 28.0 |  |
| 40 | 4/24 | |  |  |  |
| 41 | 4/30 | | + | 34.3 |  |
| 42 | 5/7 | | + | 26.8 | 0.0 |
| 43 | 5/14 | |  |  | 0.0 |
| 44 | 5/22 | |  |  | 0.0 |
| 45 | 5/28 | |  |  | 2.0 |
| 46 | 6/4 | |  |  | 22.6 |
| 47 | 6/11 | |  |  | 4.5 |
| 48 | 6/18 | |  |  | 29.6 |
| 49 | 6/24 | |  |  | 12.1 |
| 50 | 7/1 | | + | 30.9 | 56.8 |
| 51 | 7/9 | | + | 31.5 | 246.8 |
| 52 | 7/16 | | + | 26.7 | 118.3 |
| 53 | 7/23 | |  |  | 134.0 |
| 54 | 7/30 | | + | 32.2 | 22.5 |
| 55 | 8/6 | | + | 24.2 | 48.0 |
| 56 | 8/13 | | + | 31.2 | 56.8 |
| 57 | 8/27 | | + | 33.7 | 11.4 |
| 58 | 9/3 | |  |  | 24.5 |
| 59 | 9/10 | | + | 32.2 | 4.5 |
| 60 | 9/17 | | + | 26.0 | 3.3 |

*Blacklight traps are deployed on May 1 by the Rutgers Cooperative Extension; therefore, no data on April brown marmorated stink bug densities are available.
